# Comparative study of Peroneus longus tendon autograft versus Hamstring tendon autograft in arthroscopic anterior cruciate ligament reconstruction

**DOI:** 10.1007/s00264-025-06468-6

**Published:** 2025-03-07

**Authors:** Mohamed Hussein Khalil, Sherif Hamdy Zawam

**Affiliations:** Cairo University Hospitals, Cairo, Egypt

**Keywords:** Anterior cruciate ligament reconstruction, Peroneus longus autograft, Hamstring autograft

## Abstract

**Purpose:**

The purpose of this study is to compare the functional outcomes and donor site morbidities after anterior cruciate ligament reconstruction (ACLR) using peroneus longus (PL) tendon versus hamstring (HST) tendon autografts.

**Methods:**

The PL tendon autograft was used for ACLR in 36 patients, and in another group, ACLR was performed using the HST tendon autograft in 35 patients between September 2022 and April 2023. The knee functional outcomes were evaluated using the International Knee Documentation Committee (IKDC) and the Lysholm scores at preoperative and at 18 months following ACLR. In addition, the autograft diameter was measured intraoperatively in both groups. Ankle joint donor site morbidities were estimated using the American Orthopedic Foot and Ankle Score (AOFAS) in the PL autograft group.

**Results:**

A total of 71 patients, who underwent ACLR, were assessed with a minimum follow-up of 18 months (range 18–20 months). The diameter of the PL tendon autograft was significantly greater than that of the HST tendon autograft (*P* < 0.001). No significant differences were found in the functional outcomes between both groups at 18 months follow-up. Evaluation of the AOFAS showed no significant ankle joint dysfunction in the PL tendon autograft group.

**Conclusions:**

PL tendon autograft can be used as a safe and effective autograft choice for ACLR with excellent functional outcomes comparable to HST tendon autograft and minimal donor site morbidity.

**Level of evidence:**

Level II, Prospective randomized comparative study.

## Introduction

Anterior cruciate ligament (ACL) injuries account for approximately 50% of knee injuries [1]. Anterior cruciate ligament reconstruction (ACLR) is deemed to be the gold standard treatment to regain knee stability and decrease the hazards of meniscal injuries and symptomatic osteoarthritis [2].

The most frequently used autografts in ACLR encompass hamstring (HST) tendons (semitendinosus and gracilis) [3], bone-patellar tendon-bone (BPTB) [4], and quadriceps tendon [5]. Each of these autografts has advantages and drawbacks, however, there is no global consensus on autograft choice in the treatment of ACL injuries [6].

The HST tendon autografts are relatively unchallenging to harvest with little donor site morbidity, and have a tensile strength approaching that of the native ACL [7]. Nevertheless, inconstant graft size, risk of saphenous nerve paresthesia, and a possible diminish in hamstring muscle strength [7, 8] are considered HST tendon autograft drawbacks.

The peroneus longus (PL) tendon was advocated as an alternative autograft choice for ACLR [9]. The PL tendon autograft has several merits including sufficient graft size, and evaluation of its mechanical properties showed that it has appropriate strength for knee ACLR [10, 11].

There are studies in the literature comparing the functional outcomes of the PL tendon and other autografts following ACLR [12–14]. The aim of the present study is to compare the clinical outcomes and the donor site morbidities between the PL and HST autografts following ACLR. We hypothesize that the PL tendon autograft is a successful alternative to the HST tendon autografts for ACLR with comparable functional outcomes.

## Materials and methods

This study is a single-centre prospective randomized study carried out in a tertiary trauma center between September 2022 and April 2023. The research had ethical Committee Approval which was granted by the Ethics Committee of Cairo University (Institutional Review Board (IRB) number: N-1-2024). All patients signed an informed consent before joining this study. The random assignment of all patients to enter either group was computerized using simple randomization. This study began including 76 patients who underwent ACLR using PL autograft (38 patients) in group A and HST autograft (38 patients) in group B. A total of five patients (two in the PL group and three in the HST group) were excluded from the study results as they had ACL re-ruptures (following traumatic events during contact sports) before the completion of 18 months follow-up. ACL re-ruptures occurred at nine months after ACLR in two patients (one in the PL group and one in the HST group), at 12 months in two patients in the HST group, and at 14 months in one patient in the PL group. No failure of fixation was noted in any patient with ACL re-ruptures. Therefore, a total of 71 patients were involved in this study who completed the 18 months follow-up (PL group included 36 patients while the HST group comprised 35 patients). The harvested autograft diameter was recorded intraoperatively in all patients.

*The inclusion criteria were*

1. Skeletally mature patients diagnosed with complete ACL injuries complaining of giving way and knee instability.
2. Both genders were included in this study.

The ACL injuries were diagnosed through the patient’s medical history, physical examination, and magnetic resonance imaging (MRI) results.

*The exclusion criteria were*.

1. Multi-ligamentous knee injuries.
2. Skeletally immature patients.
3. Concomitant advanced knee joint chondral lesions.
4. Associated fracture around the injured knee or prior surgery to the involved knee joint.
5. Presence of associated ankle joint problem.
6. Revision ACL surgeries.

### Functional assessment

Functional outcomes were evaluated using the International Knee Documentation Committee (IKDC) and Lysholm scores and compared both preoperative and at 18 months follow-up. The American Orthopedic Foot and Ankle Score (AOFAS) was used to evaluate the ankle status and donor site morbidities in the PL autograft group. The minimum follow-up period for all patients in both groups was 18 months (range 18–20 months).

### Statistical methods

Data were represented statistically in the form of mean ± standard deviation (± SD), range, frequencies (patient number), and percentages when suitable. Numerical variables were compared between both groups using the Student t-test for independent samples. Comparison of categorical data was done using a Chi-square (χ^2^) test. In cases where the expected frequency was less than 5, an exact test was performed. A paired t-test was performed to compare numerical variables within the group. p value less than 0.05 was deemed statistically significant. All statistical analyses were performed using IBM SPSS (Statistical Package for the Social Science; IBM Corp, Armonk, NY, USA) release 22 for Microsoft Windows.

## Operation procedures

All patients were placed in the supine position after administration of either spinal or general anaesthesia. A tourniquet was applied to the upper thigh. Diagnostic arthroscopy was first done using standard anterolateral and anteromedial portals. Meniscal pathology, if present, was addressed either by meniscectomy or meniscus repair followed by graft harvesting.

### Peroneus longus tendon harvesting

**Fig. 1:**
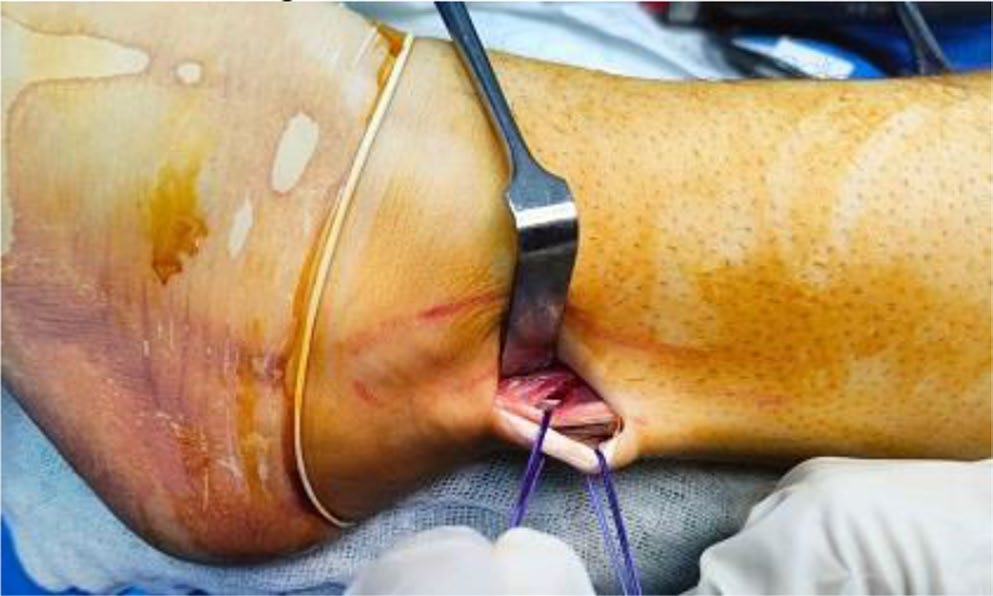
Intra-operative image showing retraction of both the PL and PB tendons. PL: Peroneus longus; PB: Peroneus brevis

**Fig. 2:**
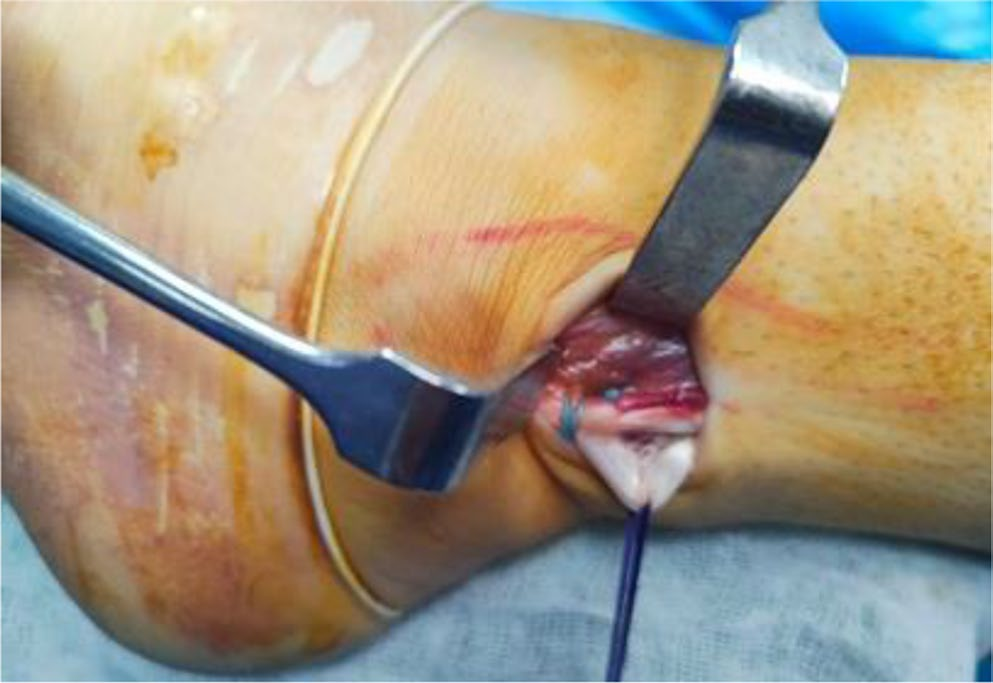
Intra-operative image showing tenodesis of both the PL and PB tendons. PL: Peroneus longus; PB: Peroneus brevis

**Fig. 3:**
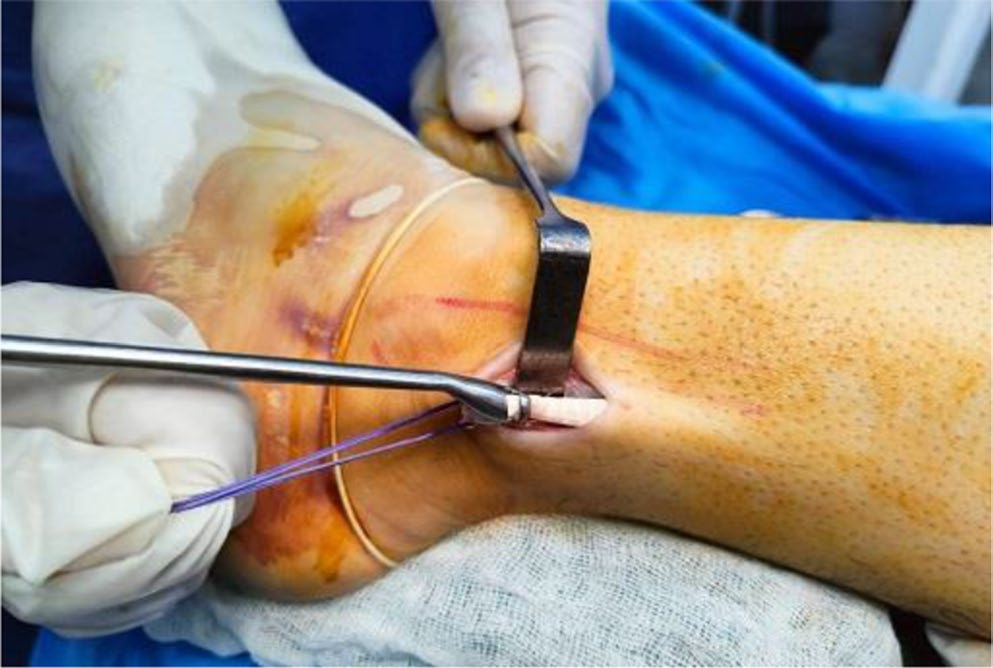
Intra-operative image showing the introduction of the tendon stripper to harvest the PL tendon. PL: Peroneus longus

**Fig. 4:**
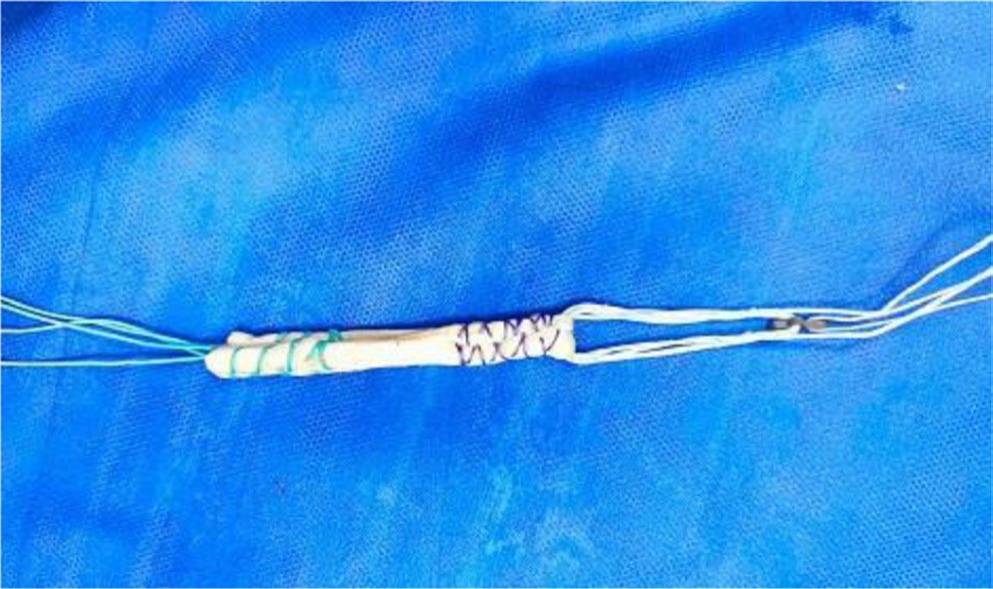
Intra-operative image showing the prepared PL autograft attached to the adjustable loop Endobutton. PL: Peroneus longus

PL tendon was harvested from the ipsilateral leg through a 2–3 cm longitudinal skin incision placed approximately 2–3 cm proximal to the posterolateral aspect of the tip of the fibula. PL and peroneus brevis (PB) tendon were dissected and delivered out of the skin incision (Fig. 1). Care was taken to avoid sural nerve injury during peroneal tendons exposure. Tenodesis of both PL and PB tendons was performed (Fig. 2). An open tendon stripper was introduced to about 4–5 cm distal to the fibular head to decrease the incidence of peroneal nerve damage during harvesting of the PL tendon (Fig. 3). The graft was doubled or tripled depending on the harvested graft length. Graft trimming was done followed by the placement of whip-stitched non-absorbable sutures at both ends of the graft. The prepared PL autograft is shown in (Fig. 4).

### Hamstring tendon harvesting

**Fig. 5:**
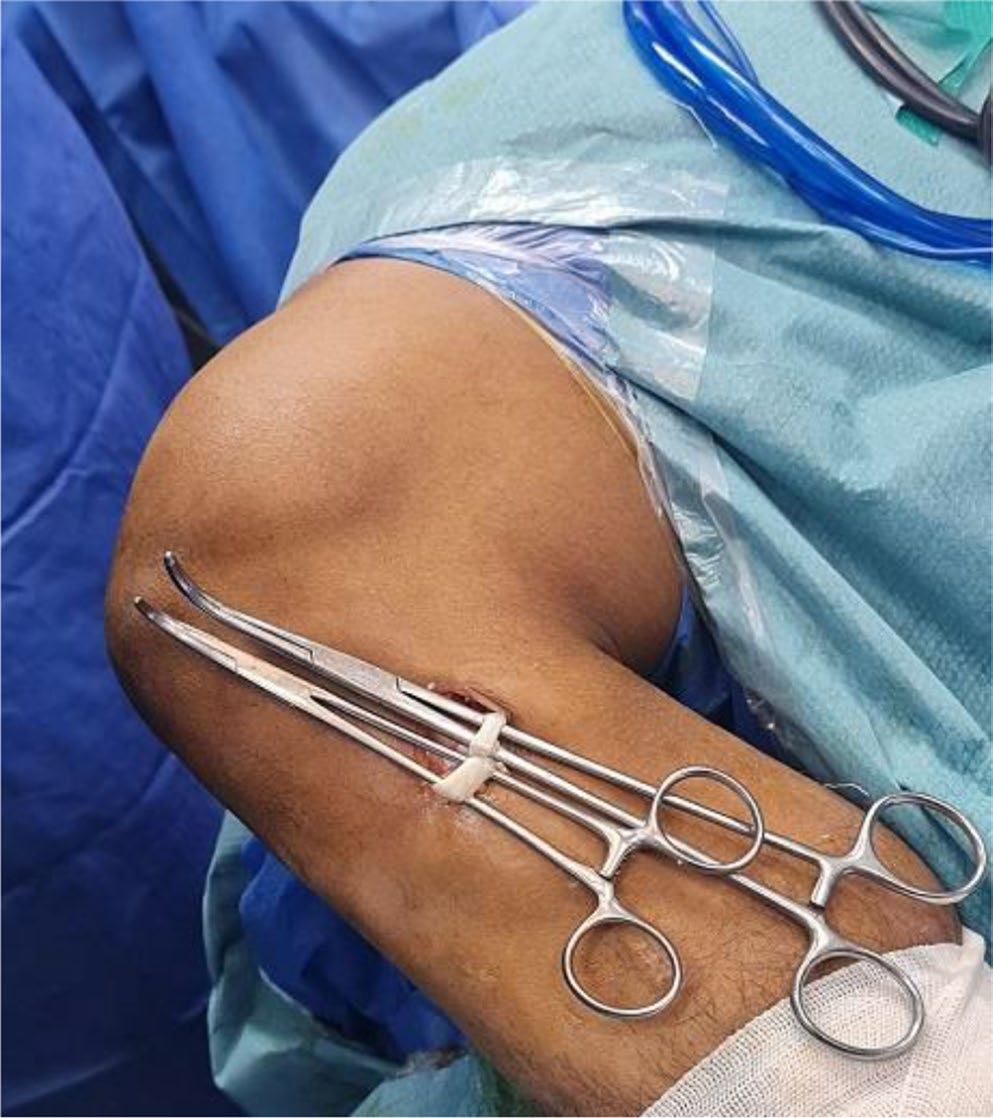
Intra-operative image showing retraction of both the semitendinosus and gracilis tendons after opening the sartorial sheath

**Fig. 6:**
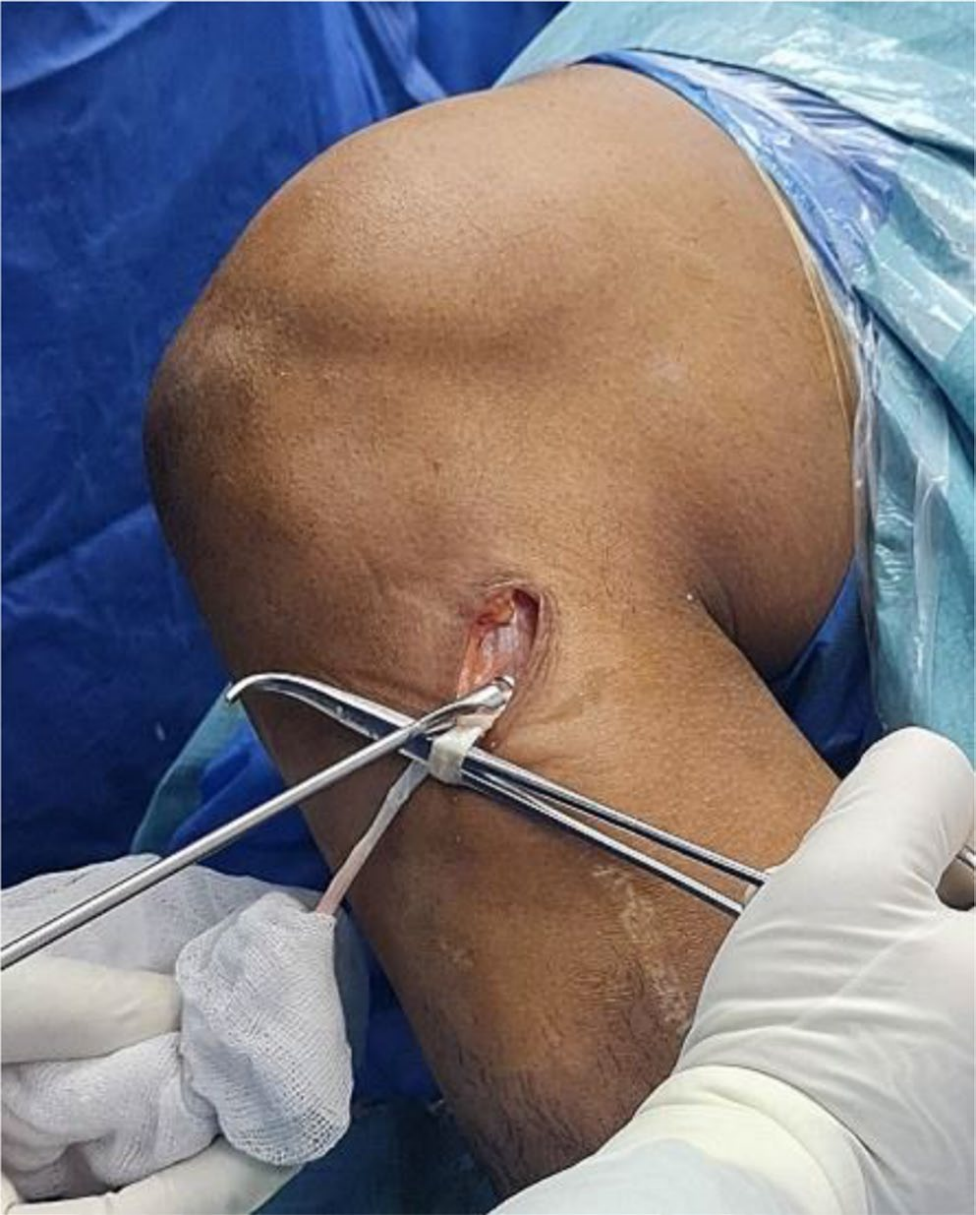
Intra-operative image showing the introduction of the tendon stripper to harvest the HST tendon. HST: Hamstring

A 3 cm oblique skin incision was done over the pes anserinus region over the anteromedial aspect of the tibia. The sartorial fascia was opened to deliver both the semitendinosus and gracilis tendons (Fig. 5). An open tendon stripper was then used to harvest both tendons (Fig. 6). The tendons were then tripled or quadrupled depending on the harvested graft length followed by placement of whip-stitched non-absorbable sutures at both ends of the graft.

### Fixation of the harvested autograft

The same intra-articular surgical technique was used in both groups. Both the femoral and the tibial tunnels were drilled independently, according to the measured autograft diameter, in anatomical position followed by passage of the prepared autograft. Femoral side fixation was achieved using an adjustable loop Endobutton. A bioabsorbable screw, one size larger than the tibial tunnel diameter, was used to fix the graft on the tibial side.

### Rehabilitation

Both groups followed the same standard postoperative protocol for ACLR. All patients started Knee extension, patellar mobility, and ankle pump exercises from day one postoperative. Full passive knee extension along with ice fomentations and prescription of analgesic anti-inflammatory medications was done in the 1st week. Closed chain quadriceps exercises in a knee brace, passive closed chain knee bending, partial weight-bearing (as pain tolerated), and a knee range of motion (ROM) of 0° to 90° were allowed in the first two weeks postoperative. Open chain quadriceps and active open chain knee bending exercises were permitted from second to fourth week postoperative.

Full flexion was obtained within five to six weeks postoperative. Full weight-bearing was allowed three to four weeks following ACLR. Quadriceps and HST exercises were continued till the third months after ACLR. Active resisted knee ROM exercises were done between the third and sixth months postoperative. Jogging and squats were allowed between the fourth and sixth months postoperative. Ankle eversion and plantarflexion strengthening exercises in the PL group were started fourweeks postoperative.

Return to sports activities in both groups was allowed only nine months after ACLR. Return to sports was permitted only for patients who had successful triple one leg hop test, satisfactory functional outcomes (IKDC score: 85 or more), had full pain-free knee joint ROM, stable knee (assessed by Lachman, anterior drawer, and pivot shift tests), and regained quadriceps girth comparable to the uninjured side.

## Results

The present study included 71 patients (completed 18 months follow-up), who underwent ACLR using either the PL autograft (36 patients) in group A or the HST autograft (35 patients) in group B. The present study included 65 male patients and six female patients. The right knee was injured in 38 patients, while the left knee was involved in 33 patients. The mean age of patients at the time of surgery in group A and group B were 27.89 ± 3.37 and 27.57 ± 4.22 years respectively. The mean follow-up period for group A was 18.36 ± 0.68 months, while the mean follow-up period for group B was 18.46 ± 0.7 months. No statistically significant differences were found between both groups regarding the demographic data and associated knee injuries (p-value > 0.05) [Table 1].

**Table 1:** Demographic data of both the PL and the HST groups

	PL *n* = 36	HST *n* = 35	*p*-value
Age	27.89 ± 3.37 (20–39)	27.57 ± 4.22 (21–38)	0.73
Gender			1
Male	33 (91.7%)	32 (91.4%)	
Female	3 (8.3%)	3 (8.6%)	
Affected knee side			0.55
Right	18 (50%)	20 (57.1%)	
Left	18 (50%)	15 (42.9%)	
Time interval between injury and surgery in months	6.39 ± 2.81 (3–12)	6.86 ± 2.8 (3–12)	0.48
Follow-up period in months	18.36 ± 0.68 (18–20)	18.46 ± 0.7 (18–20)	0.56
Mean preoperative IKDC score	56.9 ± 5.08 (43.7–69)	55.76 ± 3.3 (43.7–62.1)	0.27
Mean preoperative Lysholm score	58.89 ± 8.41 (39–71)	59.6 ± 7.85 (39–71)	0.71
Associated knee injuries Isolated ACL rupture Medial meniscus tear (repair) Lateral meniscus tear (repair) Meniscus tear (partial meniscectomy)			0.93
	19 (52.8%)	20 (57.1%)	
	7 (19.4%)	6 (17.1%)	
	6 (16.7%)	7 (20%)	
	4 (11.1%)	2 (5.8%)	

IKDC: International Knee Documentation Committee; PL: Peroneus longus; HST: Hamstring; n: number of patients. Values are expressed in the form of mean ± standard deviation (SD), range, number of patients, and their percentage within the group

The mean autograft diameter in the PL group was 8.64 ± 0.49, while the mean diameter of the HST autograft was 7.63 ± 0.55, and that showed a statistically significant difference between both groups (p-value < 0.001) [Table 2].

**Table 2:** Comparison of graft diameter between PL and HST groups

	Diameter (mm)	*p*-value
PL group (*n* = 36)	8.64 ± 0.49 (range 8–9 mm)	(*P* < 0.001)
HST group (*n* = 35)	7.63 ± 0.55 (range 7–9 mm)	

PL: Peroneus longus; HST: Hamstring; n: number of patients. Values are expressed in the form of mean ± standard deviation (SD), range

### Functional outcomes

Functional outcomes in both groups are shown in (Table 3). The mean IKDC score in group A improved significantly from 56.9 ± 5.08 preoperative to 92.78 ± 4.09 at 18 months postoperative (p-value < 0.001), while in group B, it ameliorated from 55.76 ± 3.3 to 92.31 ± 3.74 (p-value < 0.001). The mean Lysholm score in group A improved significantly from 58.89 ± 8.41 preoperative to 88.14 ± 4.89 at 18 months follow-up (p-value < 0.001), while in group B, it ameliorated significantly from 59.6 ± 7.85 to 87.83 ± 4.62 respectively (p-value < 0.001). However, no statistically significant differences were found in the context of the IKDC and Lysholm scores between both groups 18 months following ACLR.

**Table 3:** Comparison of functional outcomes between PL and HST groups

		Preoperative	18 months follow-up	*p*-value
IKDC score	PL group (*n* = 36)	56.9 ± 5.08	92.78 ± 4.09	(p-value < 0.001)
	HST group (*n* = 35)	55.76 ± 3.3	92.31 ± 3.74	(p-value < 0.001)
	P value	0.27	0.62	
Lysholm score	PL group (*n* = 36)	58.89 ± 8.41	88.14 ± 4.89	(p-value < 0.001)
	HST group (*n* = 35)	59.6 ± 7.85	87.83 ± 4.62	(p-value < 0.001)
	P value	0.71	0.78	

IKDC: International Knee Documentation Committee; PL: Peroneus longus; HST: Hamstring; n: number of patients. Values are expressed in the form of mean ± standard deviation (SD)

The mean AOFAS score for the donor’s ankle in the PL group at 18 months follow-up was 99.11 ± 3.06 (range 87–100; Excellent = 90–100 points, Good = 75–89 points, Fair = 60–74 points, and Poor < 60 points), and there were no significant differences compared the contralateral healthy ankles (p-value > 0.05). Excellent AOFAS scores were observed in 34 (94.4%) patients in the PL group 18 months following ACLR.

### Knee stability

Knee joint stability was evaluated in both groups using the Lachman, anterior drawer, and pivot shift tests and compared both preoperative and at 18 months follow-up (Table 4).

**Table 4:** Comparison of knee stability between PL and HST groups

	PL Group (*n* = 36)		HST Group (*n* = 35)		*p*-value
	*n*	%	*n*	%	
**Anterior drawer test**					
Preoperative					1
Grade 0	0	0	0	0	
Grade 1	0	0	0	0	
Grade 2	5	13.9	4	11.4	
Grade 3	31	86.1	31	88.6	
18 months follow-up					0.96
Grade 0	30	83.3	29	82.9	
Grade 1	6	16.7	6	17.1	
Grade 2	0	0	0	0	
Grade 3	0	0	0	0	
**Lachman test**					
Preoperative					1
Grade 0 (< 2 mm)	0	0	0	0	
Grade 1 (3–5 mm)	0	0	0	0	
Grade 2 (6–9 mm)	4	11.1	3	8.6	
Grade 3 (> 10 mm)	32	88.9	32	91.4	
18 months follow-up					0.73
Grade 0 (< 2 mm)	27	75	25	71.4	
Grade 1 (3–5 mm)	9	25	10	28.6	
Grade 2 (6–9 mm)	0	0	0	0	
Grade 3 (> 10 mm)	0	0	0	0	
**Pivot shift test**					
Preoperative					
Negative	0	0	0	0	
Positive	36	100	35	100	
18 months follow-up					
Negative	36	100	35	100	
positive	0	0	0	0	

PL: Peroneus longus; HST: Hamstring; n: number of patients. Values are expressed in the form of number of patients and their percentage within the group

### Complications

Several complications have been encountered in this study. Superficial wound infection was reported in two (5.6%) patients in the PL group and three (8.6%) patients in the HST group at the donor site which resolved completely with repeated wound dressing and oral antibiotics. Two (5.6%) patients in the PL group complained of mild paraesthesia and dysaesthesia at the donor site. Donor ankle morbidities following PL harvesting were evaluated using the AOFAS score. None of the patients in this study had postoperative ankle joint dysfunction or difficulties in practicing sports as a consequence of PL tendon harvesting. Five patients (14.3%) in the HST group had paraesthesia at the upper medial aspect of the leg, due to irritation of the infrapatellar branch of the saphenous nerve, which responded to conservative treatment. No statistically significant differences were observed between both groups regarding the overall rate of complications (p-value > 0.05).

## Discussion

The type of harvested graft for ACLR is usually according to the surgeon’s preferences. Preoperative autograft choice for ACLR depends on autograft size, strength, donor site complications, abundance, and patient activity level [11]. Rudy et al. proved in a biomechanical study that no significant differences were observed regarding the tensile strength between both the PL and HST tendons autografts [15].

The PL tendon autograft was documented in several studies to have good results following ACLR, regarding both functional outcomes and knee stability [12–14]. The principal finding in our study is that a significant improvement in the functional scores (Lysholm and IKDC subjective scores) was observed between preoperative and 18 months following ACLR in both groups. Nevertheless, no significant differences in the context of functional scores at 18 months follow-up were found between both groups after ACLR.

The present study results are in line with a study by Rhatomy et al. who concluded that there were no significant differences between PL and HST groups at one year following ACLR in terms of the IKDC, modified Cincinnati, and Lysholm scores [12].

A systematic review by Quinn et al. reported no significant differences in the functional outcomes between PL tendon and HST tendon autografts following ACLR with minimal morbidities at the donor site following PL tendon harvesting [13].

A study by Wu et al. including 56 patients, who underwent ACLR using PL autograft in one group and HST autograft in another group, proved that the Lysholm and the IKDC scores in the PL group were significantly better than those of the HST group at 6 months following ACLR. However, no significant differences were found in terms of the functional scores between both groups at two years following ACLR [14].

A recent study by Agarwal et al. including 194 patients, comparing the functional outcomes following ACLR using PL and HST autografts, reported no significant differences between PL and HST autografts in terms of the IKDC and Lysholm scores at six months and one year following ACLR [16].

However, a systematic review and meta-analysis including 925 patients showed that patients who underwent ACLR using the PL tendon autograft had significantly better Lysholm and IKDC scores compared to those who were treated using the HST tendon autograft. In addition, no significant differences were observed between both autografts in terms of the Tegner activity scale, Lachman test, donor site pain, or graft failure. Finally, the Foot and Ankle Disability Index (FADI) showed no significant differences, while the AOFAS score was significantly reduced at the final follow-up compared to preoperative values in the PL group [17].

A prospective comparative study documented that the PL tendon autograft showed significantly better modified Cincinnati scores compared to the HST tendon autograft at one year follow-up following ACLR with no significant differences between both autografts regarding the Lysholm score at one year follow-up. However, knee flexion strength was significantly higher in the PL group compared to the HST group at six and 12 months after ACLR. Although, the AOFAS score was 96.43 ± 3.13 at one year follow-up, none of the patients in the PL group had decreased ankle ROM or ankle instability [18].

In the present study, no significant pain or donor site complications were reported following PL tendon harvesting and 34 patients (94.4%) in the PL group achieved excellent AOFAS scores. In addition, no significant differences were found between the AOFAS scores at the donor site compared to the contralateral sound ankles at 18 months follow-up. This may be attributed to the fact that the PB muscle is a more strenuous ankle evertor than PL and remains to be after harvesting the PL tendon as mentioned by Otis et al. in their study [19].

Furthermore, Kerimoğlu et al. stated that the PL tendon plays a minor role in carrying the arch of the foot [20]. The results of the AOFAS scores at 18 months follow-up in our study are in line with those reported by Agarwal et al. who reported a mean AOFAS score of 99.05 ± 3.56 atone year follow-up in the PL group with no significant differences between the PL and HST groups following ACLR [16].

Wiradiputra et al. showed that the PL tendon harvesting autograft had no obvious postoperative morbidity associated with biomechanical inconveniency to the donor site following ACLR [21]. In addition, a study by Rhatomy et al. showed that harvesting the PL tendon had a minor influence on the foot and ankle function [22]. However, Angthong et al. reported reduced peak torque eversion and inversion, decreased ankle function, and concerns about ankle stability following PL tendon harvesting for ACLR [23].

A large cohort study of patients by Snaebjornsson et al. stated that a 0.5 mm increase in graft diameter decreased the incidence of re-operation following ACLR by 0.86 times [24]. In our study, the diameter of the PL autograft was significantly larger than that of the HST autograft (p-value < 0.001). However, our study showed that, despite the greater diameter in the PL autograft group, the incidence of ACL re-injuries showed no significant differences between PL and the HST groups (p-value > 0.05).

### Limitation

This study has some limitations. First, is the relatively short follow-up period. Second, is that patients were operated on by the two main authors, however, the same surgical steps were precisely followed in all patients. Finally, the lack of assessment of the knee flexion strength in the HST group or the ankle eversion strength in the PL group using a dynamometer. However, Authors relied on using the AOFAS score for assessment of ankle joint status as it includes evaluation of the pain, function, and alignment of the ankle joint. In addition, the IKDC and Lysholm scores were used in this study for evaluation of the knee joint function, while knee stability was evaluated clinically using the Lachman, anterior drawer, and pivot shift tests. Furthermore, several studies in literature compared the PL and the HST autograft for ACLR without muscle strength assessment [12, 16, 25].

## Conclusion

PL tendon autograft can be used as a safe and effective autograft choice for ACLR with excellent functional outcomes (based on IKDC and Lysholm knee scores) comparable to HST tendon autograft with minimal donor site morbidity.

## Data Availability

No datasets were generated or analysed during the current study.

## Abbreviations

ACL: Anterior cruciate ligament
ACLR: Anterior cruciate ligament reconstruction
AOFAS: American Orthopedic Foot and Ankle Score
BPTB: Bone-patellar tendon-bone
HST: Hamstring
IKDC: International Knee Documentation Committee
PB: Peroneus Brevis
PL: Peroneus longus
ROM: Range of motion
SD: Standard deviation
SPSS: Statistical Package for the Social Science
FADI: Foot and Ankle Disability Index
